# Effect of direct-fed microbial feed blocks on blood β-hydroxybutyrate and milk yield in early postpartum buffaloes under field conditions

**DOI:** 10.14202/vetworld.2026.440-447

**Published:** 2026-01-31

**Authors:** Duggirala Srinivas Murty, Vishal Suthar, Bhavinaben Manharbhai Rathva, Manasvi Bhikhabhai Ladola, Aarti Bipinbhai Desai, Raj Desai, Deepak B Patil, Paresh Pandya

**Affiliations:** 1 Department of Microbiology, Gujarat Vidyapith, Ahmedabad- 380014, Gujarat, India; 2 Animal Biotechnology Division, Gujarat Biotechnology University, GIFT City, Gandhinagar-382355, Gujarat, India; 3 Directorate of Research, Kamdhenu University, Gandhinagar-382010, Gujarat, India; 4 Animal Nutrition Research Station, Anand-388001, Gujarat, India

**Keywords:** beta-hydroxybutyrate, buffalo, direct-fed microbials, early postpartum, hyperketonemia, metabolic health, milk yield, probiotic feed

## Abstract

**Background and Aim:**

Early postpartum buffaloes are highly susceptible to negative energy balance and hyperketonemia, which adversely affect metabolic health, milk yield, and farm profitability under smallholder field conditions. Direct-fed microbials (DFMs) have emerged as a promising nutritional strategy to improve rumen function and productivity; however, evidence in buffaloes under real-farm settings remains limited. This study evaluated the effect of DFM feed blocks on blood β-hydroxybutyrate (BHB) concentrations and milk yield in early postpartum buffaloes managed at farmers’ doorsteps.

**Materials and Methods:**

A field-based randomized controlled trial was conducted between February and December 2023 in three dairy herds in Gandhinagar district, Gujarat, India. Initially, 36 early postpartum buffaloes were enrolled; due to attrition, 22 animals (11 per group) were included in the final analysis. Buffaloes were randomly allocated to a DFM group or a placebo group. The DFM group received feed blocks containing a consortium of probiotic strains cultivated on agricultural by-products, while the placebo group received wheat straw blocks, both alongside a nutritionally balanced basal diet. The intervention lasted five weeks. Blood BHB concentrations were measured weekly on days 0, 7, 14, 21, and 28 using a validated handheld meter, and daily milk yield was recorded throughout the study. Data were analyzed using linear mixed models with repeated-measures.

**Results:**

DFM supplementation significantly reduced mean blood BHB concentrations compared with the placebo (1.04 ± 0.04 vs. 1.40 ± 0.03 mmol/L; p < 0.05), indicating improved metabolic status. A significant treatment × time interaction demonstrated a progressive decline in BHB levels from day 7 onward in the DFM group. Buffaloes receiving DFM produced significantly more milk than controls (9.27 ± 2.91 vs. 7.35 ± 0.31 L/day), corresponding to an average increase of 1.73 ± 0.42 L/day (p < 0.001), with consistent effects across the experimental period.

**Conclusion:**

Under practical field conditions, DFM feed blocks effectively improved metabolic health by lowering blood BHB concentrations and significantly enhanced milk production in early postpartum buffaloes. These findings support the use of DFMs as a sustainable nutritional intervention for improving productivity and metabolic resilience in buffalo-based dairy systems, while highlighting the need for further mechanistic and long-term studies.

## INTRODUCTION

Although agricultural by-products and cereal crop residues constitute a substantial proportion of the diets of lactating ruminants, these feed resources are generally low in nutritive value and poorly digestible due to lignocellulosic bonds, high silica content, and the presence of anti-nutritional factors. These limitations not only constrain productive performance but also adversely affect farm profitability. Incorporating feed additives, including probiotics, prebiotics, nutraceuticals, and enzymes, represents a practical nutritional strategy to enhance feed digestibility and economic returns. The rumen hosts a complex and dynamic microbial ecosystem that is fundamental to nutrient digestion and metabolic regulation in ruminants. In this context, direct-fed microbial (DFM) supplementation, comprising beneficial bacteria, fungi, and yeasts, has gained considerable attention for its ability to modulate the rumen microbiome, improve nutrient utilization, strengthen immune responses, and enhance overall animal performance. Accumulating evidence indicates that DFMs can increase milk yield, improve milk composition, and mitigate metabolic disorders such as subacute ruminal acidosis [1–3]. In line with these benefits, our research group has recently reported encouraging outcomes using agricultural by-products as substrates in DFM formulations [4, 5].

Hyperketonemia is a metabolic disorder characterized by elevated circulating ketone body concentrations and is a major health challenge for high-producing dairy animals during the transition period. Previous studies have reported prevalence ranging from 6% to 34% in cattle, while in Indian production systems, prevalence has been estimated at 5.3% to 24.0% [6–10]. This metabolic disturbance is commonly associated with reduced feed intake and negative energy balance (NEB), which can subsequently impair milk yield, reproductive efficiency, and disease resistance. Although pharmacological and nutritional interventions have been used to control hyperketonemia, the development of sustainable, long-term preventive strategies remains an active area of research. The gut microbiome, a highly complex consortium of microorganisms, plays a pivotal role in ruminant metabolic health, and emerging evidence highlights a close link between alterations in gut microbial communities, hyperketonemia, and related metabolic disorders.

Despite growing evidence supporting the beneficial effects of DFMs on rumen function, milk production, and overall health in dairy cattle, several critical knowledge gaps remain, particularly in buffalo-based production systems and under real-world field conditions. Most existing studies have focused on controlled experimental settings, high-input dairy cattle systems, or short-term performance indicators, with limited attention to buffaloes reared by smallholder farmers. Moreover, although DFMs are increasingly promoted to improve productivity, their role in modulating metabolic disorders such as hyperketonemia during the early postpartum period remains poorly understood. In addition, the use of agricultural by-products as substrates for DFM formulation, although attractive from a sustainability and cost-effectiveness perspective, has not been sufficiently evaluated for their impact on metabolic health indicators such as blood β-hydroxybutyrate concentrations. Consequently, there is a lack of field-based evidence linking DFM supplementation with both metabolic resilience and milk yield responses in early-lactating buffaloes.

In light of these gaps, the present study was conducted to evaluate the effects of DFM feed blocks formulated from agricultural by-products on blood β-hydroxybutyrate concentrations and milk production in early postpartum buffaloes under farmers’ field conditions. Specifically, the study aimed to assess whether DFM supplementation could reduce hyperketonemia-related metabolic stress and enhance milk yield during the critical transition period. By generating field-relevant evidence, this study seeks to contribute to the development of sustainable, practical, and nutritionally sound strategies for improving metabolic health and productivity in buffalo-based dairy systems.

## MATERIALS AND METHODS

### Ethical approval

The study protocol, experimental design, and all animal-related procedures were reviewed and approved (KU/DR/IAUC/0725/2022) by the Institutional Animal Ethics Committee of Kamdhenu University, Gandhinagar, Gujarat, India. The approval covered field-based nutritional intervention studies in large ruminants and confirmed compliance with the guidelines for the care and use of animals in research prescribed by the University and relevant national regulatory authorities.

The trial was conducted under field conditions using client-owned early postpartum buffaloes, and no experimental surgery, invasive sampling, or procedures causing pain, distress, or long-term harm were performed. All interventions involved routine nutritional supplementation through DFM feed blocks and standard herd management practices commonly applied in commercial dairy systems.

Blood sampling for β-hydroxybutyrate measurement was performed by trained veterinarians using standard coccygeal venipuncture techniques, following established veterinary clinical protocols to minimize animal discomfort and stress. Body condition scoring and milk yield recording were carried out using non-invasive observational methods. Animals were monitored regularly throughout the study period for health status, feed intake, and general well-being, and no adverse effects related to the dietary intervention were observed.

Prior to enrollment, all buffaloes underwent routine health screening and deworming, and animals with clinical illness or infectious diseases were excluded to ensure animal welfare and data integrity. No antibiotics or prohibited substances were administered during the experimental period. Informed consent was obtained verbally from livestock owners after explaining the study objectives, procedures, and potential benefits, and participation was entirely voluntary.

The study adhered to the principles of good veterinary practice, animal welfare, and ethical conduct of research, and was carried out in accordance with national and international recommendations for on-farm animal research. All efforts were made to reduce animal numbers, refine experimental procedures, and replace invasive methods wherever possible, in line with the 3Rs principle (Replacement, Reduction, and Refinement).

### Study period and location

The study was conducted between February 2023 and December 2023 in Gandhinagar District, Gujarat, India.

### Animal selection and management

A convenience sample of Indian buffaloes (n = 36) was selected from three dairy herds located in Gandhinagar District, Gujarat, India. The animals had a median of 36 days in milk (DIM; interquartile range [IQR] = 20; range = 5–45 DIM) and a median third lactation [IQR = 2 (2–4); minimum = 2, maximum = 6]. The average daily milk yield across herds ranged from 5 to 11.0 kg/day, and herd sizes varied between 20 and 110 buffaloes. Sampling was conducted as part of routine health monitoring through the Ambulatory Clinics of Kamdhenu University. Prior to initiation of the trial, all animals were dewormed with fenbendazole (7 mg/kg; Panacur-3g; MSD Animal Health, India) and screened for major infectious diseases, including Brucellosis, tuberculosis, and Johne’s disease, using conventional diagnostic procedures; all animals tested negative. No antibiotics were administered during the three months preceding the study or throughout the experimental period. Demographic and production-related data, including calving date and lactation number, were systematically recorded using standardized data capture sheets in Microsoft Excel 365 (Microsoft Corp., Washington, USA).

### Development of DFM culture

A consortium of bacterial strains, including *Lactobacillus paracasei*, *Lactobacillus rhamnosis*, *Lactobacillus rhamnosus*, *Lactobacillus bifermentans*, *Lactococcus lactis*, *Lactobacillus acidophilus*, *Pediococcus acidilactici*, and *Bacillus coagulans*, was cultivated using agricultural vegetable waste following standard microbiological techniques. Detailed protocols for isolation, cultivation, characterization, inoculum preparation, and feed formulation are described in Indian Patent Application 202421030268 and in earlier reports [4, 11]. Individual feed cakes (~300 g) were prepared to enhance palatability and animal acceptance, coated with a molasses–starch mixture to improve flavor and aroma, air-dried for 12–14 h, and subsequently vacuum-sealed in high-density polyethylene bags to ensure freshness and shelf stability.

### Sample collection

#### Blood sampling and β-hydroxybutyrate measurement

Blood samples were collected from coccygeal vessels using an 18-gauge × 2.54 cm needle and transferred into 8 mL Vacutainers (clot-activated for serum and lithium heparin for plasma; Becton Dickinson India Pvt. Ltd., India). Samples were maintained at 18°C and transported to the laboratory within 1 h of collection. Blood β-hydroxybutyrate (BHB) concentrations were measured immediately on-site using a handheld BHBCheck meter (PortaCheck, Inc., NJ, USA), selected for its rapid response time and minimal blood volume requirement. The measurement procedure followed the validated protocol described by Suthar and Patil [10], whereby the test strip sensor was directly immersed in whole blood.

#### Body condition score (BCS)

BCS was assessed using a visual scoring system originally developed for cattle [12] and adapted for buffaloes with minor modifications. A five-point scale ranging from 1 (emaciated) to 5 (severely overconditioned), with 0.25-unit increments, was used by a trained veterinarian throughout the experimental period.

#### Milk recording

Milking was performed manually on all farms, and milk yield was measured using graduated buckets. Milk production from enrolled buffaloes was recorded weekly during both morning and evening milkings and documented in Excel spreadsheets.

### Experimental design and feeding regimen

Initially, 36 buffaloes from three farms were randomly allocated to either a treatment group (n = 18) or a placebo group (n = 18). Due to illness (n = 4), animal sales (n = 5), and missing samples (n = 3), the final analysis included 22 buffaloes, with 11 animals per group. Both groups were fed a basal diet formulated using locally available feed resources and commercial concentrate mixtures to meet maintenance and production requirements, in accordance with Indian Council of Agricultural Research guidelines [13].

The basal ration consisted of a commercial concentrate mixture from Sabar Dairy (Sabar Dan; 22% crude protein), a mineral mixture (30 g/day; Agrimin fort®, Virbac Pvt. Ltd., India), crushed maize, cottonseed cake, and maize cake, along with ad libitum cereal straw (paddy/maize/wheat straw) and approximately 10 kg/day of green fodder (jowar forage). The estimated nutrient composition of the basal diet on a dry matter basis was: dry matter 90% ± 2%, crude protein 12%–13%, total digestible nutrients 60%–62%, calcium 0.6%–0.7%, and phosphorus 0.35%–0.40%.

The treatment group received DFM feed blocks at 4% of the basal diet daily for five weeks, whereas the placebo group received an equivalent quantity of wheat straw blocks. Blood sampling, BCS assessment, and milk recording were conducted weekly throughout the experimental period.

### Sample size calculation

Of the 36 buffaloes initially enrolled, 22 (11 per group) completed the study. A retrospective power analysis performed using G*Power (version 3.1.9.7) for a two-tailed t-test (α = 0.05) indicated a statistical power of 87% for milk yield (Cohen’s d = 1.1) and 88% for BHB concentration (d = 1.13). The power curve suggested that a minimum of nine animals per group would be sufficient to achieve 80% power. Therefore, the final sample size (n = 11 per group) was considered adequate, retaining robust statistical power (≥85%) despite field-related losses.

### Statistical analysis

All data were recorded in a standardized format and digitized using Microsoft Excel (Microsoft Corp., Washington, USA). Statistical analyses were performed using SPSS version 26 (IBM Corp., NY, USA). Descriptive statistics were used to evaluate data distribution, sphericity, and mean values (± SE) for variables such as BHB concentrations. Normality and homogeneity of variance were assessed using the Shapiro–Wilk and Levene’s tests, respectively, and data that did not meet these assumptions were log-transformed when required.

Biologically relevant factors, including parity, weekly measurements (BHB and milk yield), BCS, farm, and their potential interactions across days 0, 7, 14, 21, and 28 (treatment × time), were analyzed using repeated-measures analysis of variance within a linear mixed model framework. Covariance structures (compound symmetry, autoregressive, and diagonal) were compared using the Akaike Information Criterion (AIC), and the diagonal structure was selected based on the lowest AIC value. Experimental buffaloes nested within treatment groups were included as random effects to account for animal- and herd-level variability, while treatment, time, and their interaction were included as fixed effects. BCS (p = 0.531) and farm (p = 0.127) showed no significant effects and were excluded from the final model. Mean differences were evaluated using Bonferroni post hoc adjustment, and statistical significance was declared at p < 0.05.

## RESULTS AND DISCUSSION

### Context of buffalo production and postpartum metabolic challenges

Compared with Europe and North America, average herd sizes in countries such as India are relatively small, and livestock production is predominantly practiced by marginal or landless farmers [14]. Despite lower individual animal productivity than in European and North American bovines, buffaloes form the backbone of the Indian dairy sector, contributing approximately 49% of total national milk production because of their high milk yield potential and the nutritional quality of their milk [15]. During the transition period, hyperketonemia is a major risk factor for postpartum disorders, including subclinical and clinical ketosis, mastitis, lameness, displaced abomasum, and metritis [7, 10]. These conditions often result in early culling and substantial economic losses for farmers. Moreover, production-related diseases are highly prevalent among Indian dairy cows and buffaloes [16].

Various therapeutic strategies have been adopted to mitigate hyperketonemia, such as oral drenching of propylene glycol (300 g/cow, per os) every 24 h for three days in mild cases, with extension up to five days in severe cases. Vitamin B12 (1.25–5 mg/cow, intramuscular) is commonly administered as an adjunct to enhance gluconeogenesis [17]. However, sustainable and farm-friendly alternatives remain limited. To the best of our knowledge, the present study is the first to evaluate the use of DFM as a strategy to manage hyperketonemia in postpartum buffaloes.

### Effect of DFM on blood β-hydroxybutyrate levels

**Figure 1:**
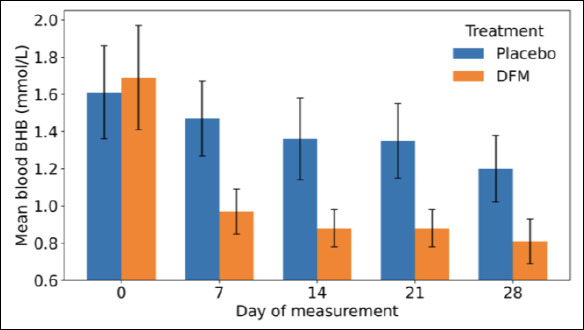
Effect of direct-fed microbials and placebo on blood β-hydroxy butyrate levels on days 0, 7, 14, 21, and 28 of enrolled buffaloes. DFM = direct-fed microbial.

Blood BHB concentrations were significantly influenced by treatment, time, and their interaction (p = 0.05, p = 0.0001, and p = 0.0001, respectively). Buffaloes supplemented with DFM exhibited significantly lower mean BHB concentrations (1.04 ± 0.04 mmol/L) compared with the placebo group (1.40 ± 0.03 mmol/L; p = 0.05), corresponding to a mean difference of 0.35 ± 0.05 mmol/L. The interaction between treatment and time of blood BHB measurement is illustrated in Figure 1, while detailed descriptive statistics for days 7, 14, 21, and 28 are presented in Table 1.

**Table 1:** Descriptive statistics showing the effect of placebo or DFM treatment on blood BHB concentrations measured using a BHB meter on days 0, 7, 14, 21, and 28 of treatment in early postpartum buffaloes.

Parameter	Day 0		Day 7		Day 14		Day 21		Day 28	
				
	Placebo	DFM	Placebo	DFM	Placebo	DFM	Placebo	DFM	Placebo	DFM
Number of buffaloes bred (n)	11	11	11	11	11	11	11	11	11	11
Mean BHB (mmol/L)¹	1.61	1.69	1.47	0.97	1.36	0.88	1.35	0.88	1.20	0.81
Median BHB (mmol/L)	1.80	1.80	1.50	1.00	1.40	1.00	1.30	0.90	1.20	0.80
Mode BHB (mmol/L)	1.80	1.80	1.50	1.00	1.00	1.00	1.10	0.90	1.00	0.80
Range BHB (mmol/L)	1.20	1.30	1.00	0.50	1.10	0.40	1.00	0.40	0.70	0.50
Minimum BHB (mmol/L)	0.90	1.10	0.80	0.80	1.00	0.60	1.00	0.70	1.00	0.50
Maximum BHB (mmol/L)	2.10	2.40	1.80	1.30	2.10	1.00	2.00	1.10	1.70	1.00
Buffaloes above HYK¹ (n)	9	9	9	3	6	2	7	0	4	0

BHB = β-hydroxybutyrate, DFM = direct-fed microbial. ¹ Hyperketonemia (HYK) defined as ≥ 1.2 mmol/L blood β-hydroxybutyrate.

From day 7 onward, buffaloes receiving DFM showed a consistent reduction in blood BHB concentrations compared with placebo-treated animals, with values frequently falling below the hyperketonemia threshold. The significant treatment × time interaction indicates notable temporal dynamics, suggesting that the effect of DFM on BHB concentrations varied over the experimental period. This reduction in circulating BHB may contribute to improved NEB and enhanced immune status in postpartum buffaloes. Although the study involved a convenience sample of relatively small size, the observed effects are encouraging and may reflect differences in geographical location, species, feeding systems, and production levels. Overall, the reduction in BHB concentrations supports the potential role of DFM in improving metabolic health during early lactation.

### Prevalence of hyperketonemia

Although assessing hyperketonemia prevalence was not the primary objective, the proportion of buffaloes with blood BHB concentrations ≥1.2 mmol/L at enrollment (day 0; 18.2%) was lower than previously reported values of 21.8%–47.2% [6, 18], within the range of 14%–19% [10, 19–21], and higher than 11% [22, 23]. Variability in prevalence may be attributed to differences in geographical conditions, nutritional management, physiological status, and production levels. Nevertheless, these findings underscore that hyperketonemia remains a relevant metabolic concern in buffaloes and warrants continued attention.

### Effect of DFM on milk production

**Figure 2:**
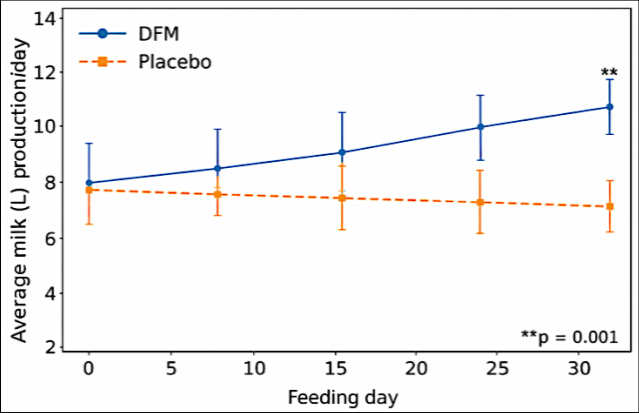
Effect of direct-fed microbials and placebo on milk production in early postpartum buffaloes on days 0, 7, 14, 21, and 28 of the experiment. DFM = direct-fed microbial.

DFM supplementation significantly affected milk production on days 0, 7, 14, 21, and 28 of the experimental period (p = 0.0001; Figure 2). No significant interaction between treatment and time of recording was observed (p = 0.188). Buffaloes receiving DFM produced significantly more milk (9.27 ± 2.91 L/day) than placebo-treated animals (7.35 ± 0.31 L/day; p = 0.0001), representing an average increase of 1.73 ± 0.42 L/day.

The magnitude of this improvement has clear practical relevance for dairy farmers and is consistent with earlier reports demonstrating positive effects of DFM on milk yield [1–3]. The absence of a treatment × time interaction suggests that the beneficial impact of DFM on milk production was stable throughout the study period and may be influenced by inherent genetic potential, nutritional adequacy, and environmental conditions. The consistent increase in milk yield is particularly important for planning long-term feeding strategies in buffalo-based dairy systems.

### Implications, limitations, and future perspectives

Despite limitations related to the short duration of the trial and variability in farm management practices, this field-based study demonstrated significant benefits of DFM supplementation in postpartum lactating buffaloes. The concurrent reduction in blood BHB concentrations and increase in milk yield highlight the potential of DFM as a practical nutritional intervention under real-farm conditions.

The encouraging outcomes warrant further investigation into the mechanisms by which DFM exerts its effects. Proposed mechanisms include modulation of the rumen microbial community structure, stabilization of rumen pH [2, 3], suppression of pathogenic microbial colonization [2, 3], improvement of energy metabolism [24], modulation of the gut–liver axis, mitigation of adverse effects associated with high-grain feeding, enhancement of rumen fermentation and nutrient absorption, and regulation of metabolic pathways [24, 25]. However, previous studies exploring these mechanisms have reported inconsistent results [2, 24, 26], likely due to differences in animal species, diets, management systems, and experimental designs. Future research incorporating targeted biochemical assessments, microbiome analyses, and refined temporal evaluations is needed to clarify these pathways and optimize the use of DFM in buffalo nutrition.

## CONCLUSION

This field-based study demonstrated that supplementation with DFM feed blocks in early postpartum buffaloes significantly improved both metabolic health and productive performance. Buffaloes receiving DFM exhibited a marked reduction in blood β-hydroxybutyrate concentrations (1.04 ± 0.04 mmol/L) compared with placebo-treated animals (1.40 ± 0.03 mmol/L), indicating alleviation of hyperketonemia and improved NEB. In parallel, DFM supplementation resulted in a consistent and significant increase in milk yield, with treated buffaloes producing an additional 1.73 ± 0.42 L/day relative to controls. These effects were maintained across the experimental period under practical farm conditions.

The observed improvements have direct relevance for buffalo-based dairy systems, particularly in smallholder and resource-limited settings. Reduction in hyperketonemia may lower the risk of postpartum metabolic and infectious diseases, potentially decreasing early culling and veterinary interventions. The associated increase in milk yield provides an immediate economic benefit to farmers, supporting the adoption of DFM feed blocks as a practical, non-pharmacological, and farm-friendly nutritional strategy during the critical transition period.

A major strength of this study is its execution under real-field conditions across multiple farms, enhancing the external validity and applicability of the findings. The use of repeated-measures, standardized sampling, and robust statistical modeling allowed reliable detection of treatment effects despite inherent farm-level variability. Additionally, the integration of metabolic (blood BHB) and production (milk yield) indicators provides a comprehensive assessment of DFM efficacy.

The study employed a convenience sample with a relatively small number of animals and a short intervention period, which may limit broader generalization of the results. Variations in farm management practices, feeding systems, and environmental conditions could also have influenced outcomes. Furthermore, mechanistic indicators such as rumen fermentation parameters, microbial community dynamics, and detailed energy metabolism markers were not assessed.

Future investigations should include larger, multi-regional trials with longer follow-up periods to validate and extend these findings. Incorporation of rumen microbiome profiling, metabolomics, and biochemical analyses would help elucidate the biological mechanisms underlying the observed reductions in β-hydroxybutyrate and improvements in milk yield. Economic analyses and dose–response evaluations would further support evidence-based recommendations for DFM use in buffalo production systems.

Overall, this study provides novel and field-relevant evidence that DFM supplementation can effectively improve metabolic resilience and enhance milk production in early postpartum buffaloes. The findings support the inclusion of DFM feed blocks as a sustainable nutritional intervention in buffalo dairy management, while highlighting the need for continued research to optimize their application and fully understand their mode of action.

## DATA AVAILABILITY

All the generated data are included in the manuscript.

## AUTHORS’ CONTRIBUTIONS

SM, PP, DB, and VS: Conceptualized and designed the study. VS, DB, and SK: Conducted the study. RD and SK: Data collection. BR, ML, and AD: Laboratory analyses. ML, AD, and SM: Prepared the DSM feed blocks. VS, PP, and SK: Drafted the initial manuscript. DB: Critically reviewed and edited the manuscript for important intellectual content. All authors contributed to the interpretation of data, reviewed the manuscript, and approved the final version for publication. All authors agree to be accountable for all aspects of the work in accordance with ICMJE authorship guidelines.
